# Supplementary material for: Pathophysiological Significance of Neutrophilic Transfer RNA-Derived Small RNAs in Asymptomatic Moyamoya Disease
Source: Cells. 2021 May 1;10(5):1086. doi: 10.3390/cells10051086 (PMC8147334; doi:10.3390/cells10051086)

2D Structure	Local AU	Position

203 218

8mer-1a

```
5 ' - t c t c a A C A A C C A G G G a - 3 '  utr
                  |   |     |   |   |   |   |   |   |
3 ' - u g a u c U G G U G G U C C C u - 5 '  tRF
      16 15 14 13          7 6 5 4 3 2
```

3' Pairing Seed

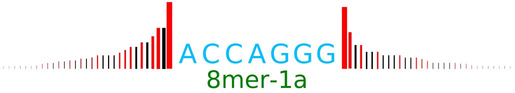

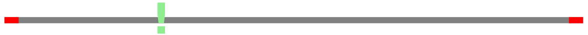

Supplement: Supplementary file 1 [file cells-10-01086-s001.zip › cells-1149161 3rd supplementary materials/Data Supplements/Data Supplement V.zip!/Data supplement 6-mRNAs targets of tsRNAs/tsRNA-Glu-CTC-003/Diagrams/tRF-Glu-CTC-003_vs_ENST00000336174(STRADA).pdf]
